# Psoriasis and gut microbes: research advances from mechanism to therapy

**DOI:** 10.3389/fmicb.2025.1711288

**Published:** 2025-12-04

**Authors:** Jianfei Chen, Keyun Sun, Xinyu Zhang, Xiaojie Chen, Yunning Chu, Limin Geng, Ziwen Bian, Yang Su, Xuefei Cong, Guoli Wang

**Affiliations:** 1 School of Traditional Chinese Medicine, Binzhou Medical University, Yantai, China; 2 Han Guang Traditional Chinese Medicine Clinic, Yantai, China; 3 Yantai Hi-tech Industrial Development Zone Hospital, Yantai, China; 4 China Institute for History of Medicine and Medical Literature, China Academy of Chinese Medical Sciences, Beijing, China

**Keywords:** gut dysbiosis, gut microbiota, gut-skin axis, immune regulation, psoriasis

## Abstract

**Background:**

Psoriasis is a chronic, immune-mediated, relapsing inflammatory skin condition, with its pathogenesis remaining incompletely understood and clinical eradication presenting significant challenges. Recent studies have highlighted the role of gut microbiota in psoriasis pathogenesis, emerging as a focal point of research.

**Objective:**

This review aims to systematically elucidate the core mechanisms by which gut microbiota contribute to psoriasis pathogenesis, summarize advances in gut microbiota-based therapeutic strategies, and provide theoretical support and innovative insights for both basic research and clinical treatment of psoriasis.

**Methods:**

Comprehensively retrieve and analyze recent research literature on the gut microbiota characteristics of psoriasis patients, the regulatory mechanisms of the gut-skin axis, and related therapeutic interventions, focusing on the microbiota’s effects on immune modulation, intestinal barrier integrity, and metabolic products.

**Results:**

Accumulating evidence supports a complex, bidirectional regulatory relationship between gut dysbiosis and skin inflammation, with notable alterations in the diversity and relative abundance of gut microbial communities in patients with psoriasis compared to healthy individuals. This review comprehensively examines the mechanisms through which gut microbes contribute to psoriasis development via the gut-skin axis, influencing immune regulation, intestinal barrier integrity, and related metabolites. Additionally, the potential of gut microbiota-based therapies—such as oral probiotics, prebiotics, synbiotics, and fecal microbiota transplantation—in alleviating psoriasis symptoms and reducing disease recurrence is emphasized.

**Conclusion:**

Dysbiosis of the gut microbiota is a key factor in the pathogenesis of psoriasis. The regulatory mechanisms of the gut-skin axis offer new insights into the multisystemic associations of psoriasis. Gut microbiota-based therapeutic strategies hold promise as important adjuncts to conventional treatments, laying the foundation for developing novel targeted therapies. This approach carries significant clinical implications for improving the prognosis of psoriasis patients.

## 1 Introduction

Psoriasis is a prevalent chronic relapsing inflammatory skin disorder, characterized by epidermal keratinocyte (KC) hyperproliferation, hyperkeratosis, tortuous dilation of dermal microvessels, and inflammatory cell infiltration. Clinically, it presents as well-demarcated red plaques covered with silvery-white scales, often accompanied by varying degrees of pruritus (Lebwohl, 2018; Boehncke and Schön, 2015). The pathogenesis of psoriasis is multifactorial, typically attributed to a combination of genetic predisposition, immune system abnormalities (particularly activation of the IL-23/Th17 pathway), and environmental factors (Vecellio et al., 2020; Griffiths et al., 2021; Alsakarneh et al., 2025). Beyond the cutaneous manifestations, patients may also experience co-morbidities, including metabolic disorders, cardiovascular diseases, inflammatory bowel disease, pulmonary infections, and psychiatric conditions (Gelfand et al., 2006; Takeshita et al., 2017; Masson et al., 2020; Hölsken et al., 2021). Despite the availability of various treatments aimed at symptom management, no method currently offers a complete cure for the disease.

The human gut harbors a complex and diverse microbial community, primarily composed of six bacterial phyla: *Firmicutes* (NCBI currently classifies it as *Bacillota*, taxid: 1239), *Bacteroidetes* (NCBI currently classifies it as *Bacteroidota*, taxid: 976), *Actinobacteria*, *Proteobacteria*, *Fusobacteria*, and *Verrucomicrobia* (Laukens et al., 2016; Polak et al., 2021). Of these, *Firmicutes* and *Bacteroidetes* dominate, comprising approximately 90% of the intestinal microbiota (Demirci et al., 2020). Growing evidence highlights the critical role of gut microbiota in regulating immune responses, nutrient metabolism, neuroendocrine functions, and defense against pathogenic microorganisms (Milani et al., 2017; Libertucci and Young, 2019; Wang et al., 2024). Physiologically, the gut microbiota plays a pivotal role in immune regulation and skin homeostasis. Dysbiosis, which disrupts the intestinal microenvironment, leads to the production of harmful metabolites, B-cell hyperreactivity, and aberrant T-cell differentiation. These disturbances can result in immune dysfunction, triggering systemic disorders, including skin inflammation (Toor et al., 2019).

The gut and skin share a high degree of vascularization, as well as similar signaling and innervation pathways, creating an optimal environment for immune and neuroendocrine functions (Mahmud et al., 2022). Recent research has progressively revealed the strong bidirectional relationship between the gut and skin. Findings highlighting the overlap of susceptibility genes between psoriasis and inflammatory bowel disease (IBD), along with the nearly doubled risk of ulcerative colitis in patients with psoriasis compared to healthy individuals, underscore the close link between psoriasis and gut dysfunction (De Francesco and Caruso, 2022). Gut microbiota and intestinal dysfunction are closely associated with inflammatory skin conditions such as psoriasis, acne, and atopic dermatitis, drawing considerable attention to the “gut-skin axis” theory (De Pessemier et al., 2021; Mahmud et al., 2022). Under normal conditions, the gut microbiota helps maintain skin homeostasis by regulating immune function. However, when dysbiosis occurs, it can compromise skin integrity and function by disrupting the gut barrier, releasing inflammatory mediators and microbial metabolites, and triggering various skin diseases (Buhaș et al., 2022; Patel et al., 2022). This review explores the changes in gut microbiota characteristics in patients with psoriasis, the mechanisms through which gut microbes influence psoriasis pathogenesis, and the progress of therapeutic strategies targeting the gut microbiota, aiming to enhance understanding of psoriasis’ pathogenesis and inform the development of novel microbiota-based treatments.

## 2 Changes in gut microbiota in psoriasis

### 2.1 Alterations in the diversity of gut microbiota in patients with psoriasis

Gut microbiota influences multiple tissues and organs, regulating immune balance through various pathways. However, it is highly susceptible to factors such as diet, age, disease, and medication. A reduction in gut microbiota diversity can contribute to immune and metabolic disorders (Schmidt et al., 2018). In this context, gut microbiota diversity primarily refers to the abundance and homogeneity of microbial species, which are commonly assessed using metrics like the number of operational taxonomic units (OTUs), alpha diversity (e.g., Shannon index, Chao1 index, ACE index), and beta diversity index. Gut microbial imbalances exacerbate psoriasis, with significant changes in the diversity and abundance of gut microbes observed in patients (van den Bogaard et al., 2021; Mahmud et al., 2022; Wen et al., 2023; Zou et al., 2024). Specifically, the Shannon index (diversity), Chao1 index (species richness), and Faith Phylogenetic diversity index of gut microorganisms have been reported to be significantly lower in patients with psoriasis compared to healthy controls, suggesting a decrease in the complexity and diversity of the microbiota (Hidalgo-Cantabrana et al., 2019). The overall diversity of fecal microorganisms in patients with new-onset, untreated psoriasis and psoriatic arthritis (PsA) is lower than in healthy individuals, with reductions in both the Shannon index and Faith Phylogenetic diversity index (Scher et al., 2015). Moreover, Huang et al. observed that while the community richness of gut microbiota was significantly lower in patients with psoriasis compared to healthy individuals, species diversity remained similar, as evidenced by reduced ACE and Chao1 indices, and unchanged Simpson or Shannon indices in patients with psoriasis (Huang et al., 2019). Despite most studies confirming reduced gut microbiota *α*-diversity in patients with psoriasis, a few studies report contrary findings. For example, some patients with psoriasis, particularly those with negative bacterial DNA translocations, exhibit a higher intestinal microbial Shannon index than healthy individuals. This may be linked to a localized inflammatory state or compensatory proliferation of specific microbiota (Codoñer et al., 2018). Additionally, while Shapiro et al. (2019) found no significant differences in alpha diversity between patients with psoriasis and healthy individuals, notable differences were observed in the functional metabolic pathways of their respective microbiota. These discrepancies may be attributed to variations in sample collection, cohort heterogeneity, and assay techniques.

Beta diversity reflects the degree of variation in microbial composition, highlighting structural differences across samples. Significant differences in beta diversity between patients with psoriasis and healthy controls have been reported, primarily due to changes in microbiota composition. Beneficial genera, such as *Akkermansia*, *Faecalibacterium*, *Blautia*, and *Parabacteroides*, are significantly reduced in patients with psoriasis, potentially linking these changes to immune dysregulation (Shapiro et al., 2019; Sikora et al., 2020; Chang et al., 2021). These findings on altered gut microbial biodiversity in psoriasis offer valuable insights into the disease’s pathogenesis and potential treatment strategies.

### 2.2 Alterations in the relative abundance of gut microbiota of patients with psoriasis

In psoriasis individuals with severe ecological dysbiosis, not only is gut microbiota diversity altered, but there is also a shift in the relative abundance of several bacterial taxa (Hidalgo-Cantabrana et al., 2019). At the phylum level, *Firmicutes* and *Bacteroidetes* dominate the gut microbiota, and the ratio of these phyla (F/B ratio) is frequently used to assess microbial imbalance. Most studies have reported an elevated relative abundance of Firmicutes and a reduced abundance of Bacteroidetes in patients with psoriasis, resulting in an increased F/B ratio (Chen et al., 2018; Hidalgo-Cantabrana et al., 2019; Shapiro et al., 2019; Xiao et al., 2021). However, a few studies have reported the opposite findings (Huang et al., 2019). Additionally, the abundance of other phyla, including *Proteobacteria*, *Actinobacteria*, *Verrucomicrobia*, and *Tenericutes*, is also altered to varying degrees in patients with psoriasis (Phan et al., 2018; Tan et al., 2018; Hidalgo-Cantabrana et al., 2019; Shapiro et al., 2019; Xue et al., 2025). At the genus level, increased relative abundance has been observed for *Bacillus*, *Blautia*, *Bifidobacterium*, *Ruminococcus*, *Streptococcus*, *Enterococcus*, *Lactococcus*, *Subdoligranulum*, and *Slackia* in patients with psoriasis, whereas the abundance of genera such as *Allobaculum*, *Alistipes*, *Coprobacillus*, *Carnobacterium*, and *Gordonibacter* is reduced (Scher et al., 2015; Tan et al., 2018; Hidalgo-Cantabrana et al., 2019; Huang et al., 2019; Shapiro et al., 2019). *Blautia*, which upregulates intestinal regulatory T cells and promotes biotransformation (Liu et al., 2021), and *Ruminococcus*, which promotes immune activation and the production of cytokines like TNF-α, suggest that immune dysregulation driven by abnormal gut microbiota may play a key role in psoriasis pathogenesis (Henke et al., 2019). However, discrepancies in the abundance of genera such as *Akkermansia*, *Bacteroides*, *Bifidobacterium*, *Faecalibacterium*, *Lachnospira*, and *Parabacteroides* require further investigation to clarify the results of different studies (Table 1) (Scher et al., 2015; Codoñer et al., 2018; Tan et al., 2018; Huang et al., 2019; Shapiro et al., 2019). At the species level, patients with psoriasis exhibit significant increases in *Collinsella aerofaciens*, *Clostridium citroniae*, *Dorea formicigenerans*, *Escherichia coli*, *Ruminococcus gnavus*, and *Akkermansia muciniphila*. Conversely, the abundance of *Faecalibacterium prausnitzii*, *Parabacteroides distasonis*, and *Prevotella copri* is decreased (Eppinga et al., 2016; Scher, 2018; Tan et al., 2018; Shapiro et al., 2019; Schade et al., 2022).

**Table 1:** Summary of gut microbiota dysbiosis in psoriasis.

References	Study group	Method of analysis	Results
Chen et al. (2018)	Psoriasis patients (*n* = 32) Healthy controls (*n* = 64)	16S rRNA V3-V4 hypervariable region	*Bacteroidetes* ↓, *Firmicutes* ↑ (phylum); *Bacteroidaceae*, *Prevotellaceae* ↓, *Ruminococcaceae*, *Lachnospiraceae* ↑ (family)
Codoñer et al. (2018)	Psoriasis patients (*n* = 52) Healthy controls (*n* = 300)	16S rRNA V3-V4 hypervariable region	*Bacteroides* ↓; *Ruminococcus*, *Faecalibacterium*, *Akkermansia* ↑ (genus)
Eppinga et al. (2016)	Psoriasis patients (*n* = 29) Healthy controls (*n* = 33)	Quantitative polymerase chain reaction	*Faecalibacterium prausnitzii* ↓; *E. coli* ↑
Hidalgo-Cantabrana et al. (2019)	Psoriasis patients (*n* = 19) Healthy controls (*n* = 20)	16S rRNA V2-V3 hypervariable region	diversity ↓, *Firmicutes* ↑, *Bacteroidetes* ↓, F/B ratio ↑, *Actinobacteria* ↑, *Proteobacteria phylum*, *Alistipes*, *Bacteroides*, *Barnesiella*, *Faecalibacterium*, *Parabacteroides* and *Paraprevotella genera* ↓
Huang et al. (2019)	Psoriasis patients (*n* = 35) Healthy controls (*n* = 27)	16S rRNA V4 and V5 region	Firmicutes ↓, *Bacteroidetes* ↑ (phylum); *Allobaculum*, *Carnobacterium*, *Gordonibacter*, *Granulicatella*, *Rothia*, *Streptococcus*, *Thermus* ↓, *Bacillus*, *Bacteroides*, *Lachnospira*, *Lactococcus*, *Parabacteroides Sutterella* ↑ (genus)
Scher et al. (2015)	Psoriasis patients (*n* = 16) Healthy controls (*n* = 17)	16S rRNA V1-V2 region and 454 pyrosequencing	*Alistipes*, *Akkermansia*, *Coprococcus*,*Pseudobutyrivibrio*, *Parabacteroides*, *Ruminococcus* ↓ (genus)
Schade et al. (2022)	Psoriasis patients (*n* = 21) Healthy controls (*n* = 24)	16S rRNA V3-V4 region	*Blautia*, *Lachnospira*, *Ruminococcus* ↓; *Catenilbacterium*, *Dialister* ↑ (genus); *Akkermansia muciniphila* ↓; *Prevotella copri* ↑
Shapiro et al. (2019)	Psoriasis patients (*n* = 19) Healthy controls (*n* = 20)	16S rRNA V4 hypervariable region	*Bacteroidetes* ↓, *Firmicutes* ↑ (phylum); F/B ratio ↑, *Prevotella* ↓, *Blautia*, *Faecalibacterium* ↑ (genus); *Ruminoccocus gnavus*, *Dorea formicigenerans*, *Collinsella aerofaciens* ↑
Tan et al. (2018)	Psoriasis patients (*n* = 14) Healthy controls (*n* = 14)	16S rRNA V4 conserved region	*Verrucomicrobia*, *Tenericutesphyla*, *Mollicutes*, *Verrucomicrobiae*, *Akkermansia muciniphil* ↓; *Bacteroides genera*, *Clostridium citroniae* spp. *Enterococcus genera* ↑
Wen et al. (2023)	Psoriasis patients (*n* = 32) Healthy controls (*n* = 32)	16S rRNA	*Firmicutes* ↓, *Bacteroidetes* ↑ (phylum); *Roseburia*, *Eubacterium* ↓ (genus); *Roseburia hominis* ↓, *Bacteroides uniformis* ↑
Xiao et al. (2021)	Psoriasis patients (*n* = 30) Healthy controls (*n* = 15)	Metagenomics sequencing	*Bacteroidetes*, *Euryarchaeota*, *Proteobacteria* ↓, *Actinobacteria*, *Firmicutes*, *Verrucomicrobia* ↑ (phylum); *Actinobacillus*, *Alistipes*, *Anaerotruncus*, *Butyricimonas*, *Comamonas*, *Eubacterium*, *Oxalobacter*, *Odoribacter*, *Pedobacter, Prevotella, Pseudoflavonifractor* ↓ *Bacteroides*, *Bifidobacterium*, *Faecalibacterium*, *Megamonas*, *Roseburia ↑ (genus)*
Zhang et al. (2021)	Psoriasis patients (*n* = 19) Healthy controls (*n* = 20)	16S rRNA	*Lachnospiraceae ↓*, *Ruminococcaceae*, *Veillonellaceae ↑ (family)*; *Megamonas*, *Faeclibacterium ↑ (genus)*

The variations in microbial diversity and specific bacterial genus abundance across different studies can be explained by the following factors. Regarding patient cohort characteristics, significant variations exist across studies in the severity and subtypes of psoriasis (e.g., plaque, pustular, erythrodermic), comorbidities (e.g., presence of metabolic syndrome or IBD), baseline dietary patterns (high-fiber/high-fat diets), and prior treatment histories (e.g., use of biologics or antibiotics). Studies have shown that patients with pustular psoriasis exhibit significantly higher abundance of *Faecalibacterium* and *Anaerorhabdus* in their gut compared to those with plaque psoriasis (Zhang et al., 2021). Furthermore, untreated patients with newly diagnosed psoriasis demonstrate markedly different gut microbiota *α*-diversity compared to those treated with immunosuppressants, potentially reflecting selective suppression of certain microbial groups by these medications (Scher et al., 2015; Codoñer et al., 2018). Furthermore, a Brazilian psoriasis cohort revealed markedly elevated abundances of *Dialister* and *Revotella copri*, while a Chinese cohort reported reduced abundances of *Roseburia* and *Eubacterium*. This discrepancy likely stems from dietary differences: the Brazilian diet’s high legume protein content may promote proliferation of *Prevotella* (which specializes in fiber degradation), whereas the Chinese diet’s high refined carbohydrate intake may reduce SCFA-producing bacteria (such as *Roseburia*) (Schade et al., 2022; Wen et al., 2023). From a methodological perspective, 16S rRNA sequencing only covers the genus level and exhibits primer bias. For instance, sequencing the V3-V4 region allows more accurate differentiation of genera within the *Ruminococcaceae* family, whereas sequencing the V4 region alone provides lower resolution for this family, detecting only family-level abundance changes without resolving genus-level differences (Chen et al., 2018; Shapiro et al., 2019). The inconsistencies in psoriasis-associated gut microbiota alterations across studies fundamentally stem from conflicting factors. Future research requires rigorously defined inclusion criteria and integration of multi-omics approaches (e.g., metagenomics, metabolomics) alongside genome-wide association studies to enhance accuracy. Despite some inconsistencies in the results across studies, the evidence clearly indicates that gut microbiota alterations are crucial to psoriasis development, and modulating the gut microbiota may offer a promising therapeutic approach for the condition.

## 3 Alterations in the diversity of gut microbiota in patients with psoriasis

### 3.1 Gut-skin axis imbalance and immune disorders

**Figure 1:**
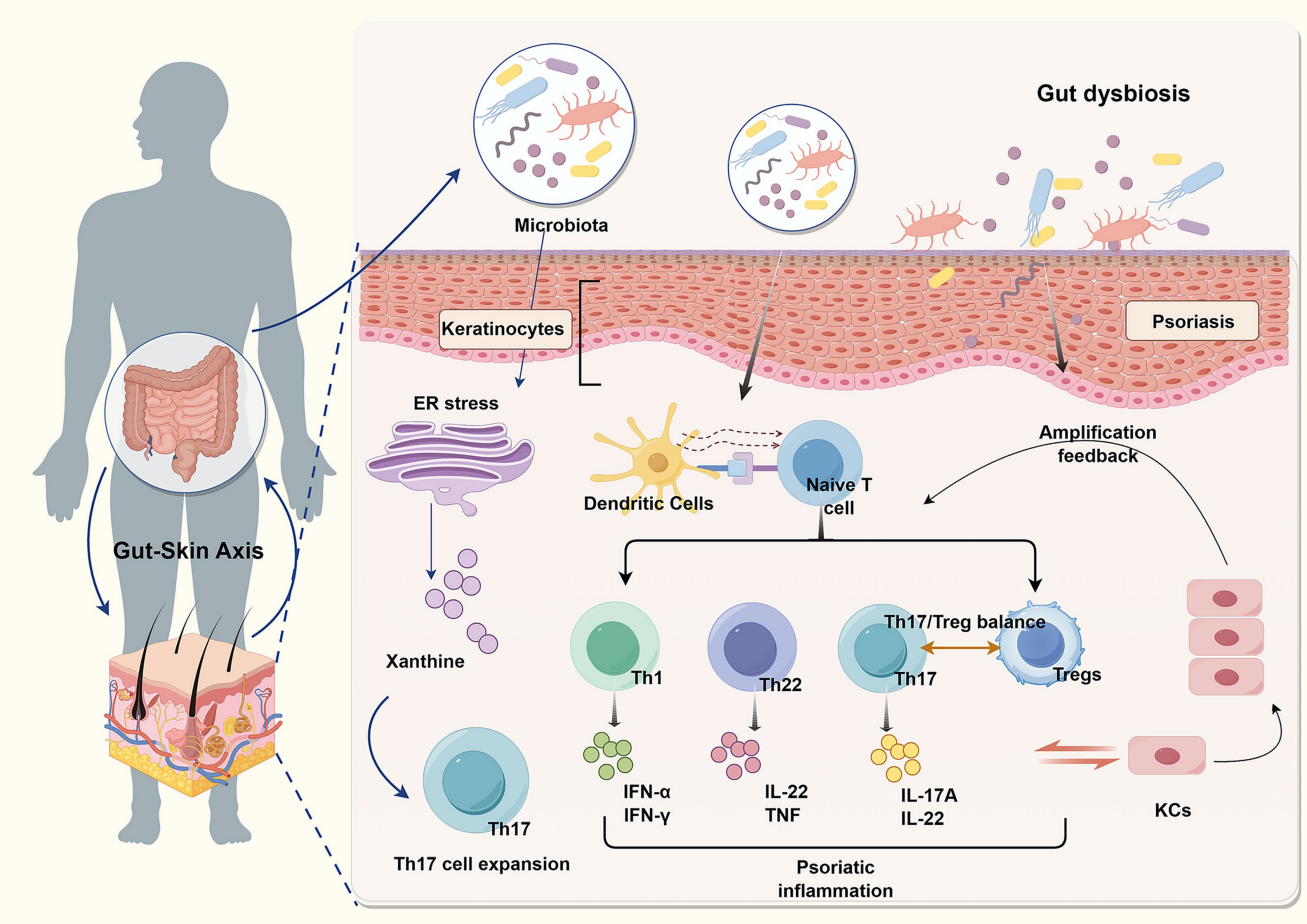
Schematic diagram of the mechanisms by which gut microbiota dysbiosis and gut-skin axis dysregulation affect psoriasis. When gut microbiota imbalance occurs, it acts on skin keratinocytes to trigger endoplasmic reticulum stress, producing xanthine and promoting Th17 cell expansion. Simultaneously, it activates skin dendritic cells, inducing the differentiation of naive T cells into Th1, Th22, and Th17 effector T cell subsets. These cells secrete cytokines such as IFN-α, IFN-γ, IL-22, TNF, and IL-17A, while disrupting the balance between Th17 cells and Tregs, thereby triggering psoriatic inflammation. This inflammation further amplifies the feedback loop, affecting T cell differentiation and keratinocytes, creating a vicious cycle that ultimately drives psoriasis progression.

The gut microbiota, often referred to as the invisible guardian of skin health, establishes a bidirectional regulatory network with the skin immune system through the gut-skin axis (Figure 1). Under physiological conditions, the gut microbiota maintains intradermal homeostasis by regulating systemic immune responses. However, dysbiosis can compromise skin integrity and function, leading to detrimental effects on skin health (Kim et al., 2020; Polkowska-Pruszyńska et al., 2020). The gut microbiota plays a pivotal role in immune dysregulation in psoriasis by influencing the differentiation, function, and intercellular signaling networks of both innate and adaptive immune cells. This regulation is bidirectional: a balanced microbiota fosters immune tolerance, while dysbiosis disrupts immune homeostasis by activating pro-inflammatory pathways (Chelakkot et al., 2018; Lin et al., 2019; Spencer et al., 2019). Despite the complex pathogenesis of psoriasis, it is well-established that the IL-23/Th17 cell axis plays a critical role in disease development, forming the foundation for the targeted biologic treatments of psoriasis used in clinical practice (Gaffen et al., 2014; Nakajima, 2012).

The gut-skin axis in psoriasis involves the regulation of T cell function and differentiation, particularly the imbalance between Treg and Th17 cells (Stehlikova et al., 2019; Zákostelská et al., 2016). Previous studies have shown that Th17 cell development in the gut is dependent on intestinal microbiota, with microorganisms such as *Clostridium*, *Bifidobacterium*, and *Ruminococcus* required to interact with intestinal epithelial cells to induce Th17 cell production (Atarashi et al., 2015; Ivanov et al., 2009). Further research has revealed that endoplasmic reticulum (ER) stress and the associated unfolded protein response (UPR) in intestinal epithelial cells may underlie the induction of Th17 differentiation by gut microorganisms. Specifically, ER stress in these cells initiates purine metabolism, which subsequently promotes Th17 differentiation in the gut (Duan et al., 2023). Overactivation of Treg/Th17 cells accelerates psoriasis progression by releasing various inflammatory factors, such as IL-23, IL-17A, IL-22, IL-6, and IFN-*α*. These cytokines drive KC proliferation and inflammation, contributing to skin pathology (Lochner et al., 2015; Guo et al., 2023; Liu et al., 2024). A study by Okada et al. (2020) confirmed that pro-inflammatory gut microbes, including *Bacteroides* spp. and *Prevotella* spp., can promote aberrant activation of Th1/Th17 cells in patients with psoriasis *via* the Toll-like receptor (TLR) signaling pathway. This leads to the overexpression of key inflammatory factors, such as IL-1β, IL-6, and TNF-α, in the skin. When skin homeostasis is disrupted, IL-15 produced by damaged KCs and IL-23 produced by dendritic cells (DCs) further stimulate inflammatory cytokines like IL-17A, IL-22, and IFN-α, which trigger KC overproliferation and skin thickening (Lowes et al., 2014). Moreover, dysbiosis of the intestinal microbiota can result in the translocation of bacteria and metabolites into the bloodstream, particularly lipopolysaccharide (LPS) released by Gram-negative bacteria. LPS activates immune cells, such as DCs and macrophages, through TLRs, triggering the psoriasis inflammatory cascade *via* the bloodstream (Traina, 2019; Xiao et al., 2021; Buhaș et al., 2022). In contrast, beneficial bacteria such as *Bifidobacterium longum* and *Lactobacillus rhamnosus* play a vital role in maintaining intestinal health by serving as the primary energy source for butyrate production. Butyrate supports gut metabolism, reduces oxidative stress, and effectively regulates microbiota homeostasis (Navarro-López et al., 2019). These findings suggest that modulating gut microbiota balance may offer a promising therapeutic strategy for managing psoriasis.

### 3.2 Gut barrier dysfunction

The integrity of the intestinal barrier is a critical link between intestinal microbiota dysbiosis and the progression of psoriasis. Disruption of the intestinal barrier leads to “leaky gut,” allowing microorganisms, their metabolites, and antigens, which are normally confined to the intestinal lumen, to enter the bloodstream and skin. This triggers a systemic inflammatory response, destabilizing skin homeostasis and potentially inducing or exacerbating psoriasis (De Francesco and Caruso, 2022; Lundquist et al., 2025). Patients with psoriasis typically exhibit increased intestinal permeability, with elevated serum levels of biomarkers such as Claudin-3, trimethylamine N-oxide (TMAO), and intestinal fatty acid binding protein (I-FABP), all of which are associated with compromised intestinal barrier function. These biomarker levels are positively correlated with the severity of psoriasis (Sikora et al., 2019; Lai et al., 2023; Stec et al., 2023). Animal studies have also confirmed a correlation between Psoriasis Area and Severity Index (PASI) scores and blood concentrations of markers of intestinal barrier damage (Langan et al., 2019). Floral metabolites, such as LPS and glycolipids, can cross the impaired intestinal barrier and enter the bloodstream. Upon entering circulation, LPS is recognized by innate immune cells, activating TLRs and triggering the inflammatory cascade characteristic of psoriasis. Glycolipids can bind to C-type lectins, further inducing immune inflammation (Schoenen et al., 2010; Buhaș et al., 2022; Kim et al., 2024). These processes are critical in triggering or exacerbating psoriasis. Furthermore, bacterial DNA, often derived from *E. coli*, has been detected in the blood of patients with psoriasis, providing additional evidence of impaired intestinal barrier function and supporting the hypothesis that intestinal bacterial translocation may be linked to psoriasis (Lao et al., 2023). Thus, intestinal barrier dysfunction and increased permeability play a central role in the pathogenesis of psoriasis, and chronic low-grade intestinal inflammation may represent a key feature of the disease.

**Figure 2:**
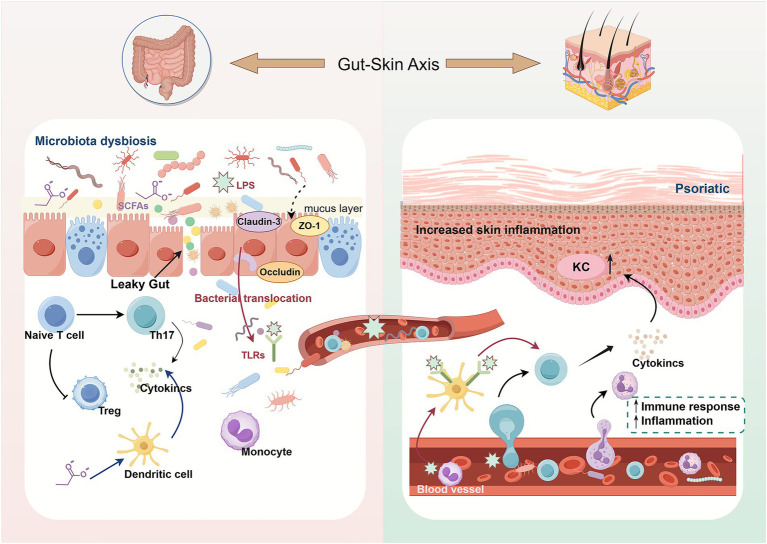
Schematic diagram illustrating the impact of impaired intestinal barrier function on psoriasis. When gut microbiota dysbiosis occurs, the tight junction proteins (Claudin-3, ZO-1, Occludin) of the intestinal barrier are compromised, leading to intestinal permeability (leaky gut). Microbiota and their metabolites (e.g., LPS, SCFAs) activate immune cells like monocytes and dendritic cells through bacterial translocation, promoting Th17 differentiation of naive T cells. This disrupts the balance with regulatory T cells (Tregs), leading to massive cytokine release. These signals are transmitted via the gut-skin axis to the skin, triggering cutaneous immune responses and inflammation. This stimulates keratinocytes (KC), exacerbates skin inflammation, and ultimately drives the development and progression of psoriatic lesions.

Impaired intestinal barrier function and dysbiosis can create a mutually reinforcing cycle. Not only does barrier dysfunction lead to dysbiosis, but dysbiosis can further exacerbate intestinal barrier impairment. In patients with psoriasis, alterations in the *Firmicutes*/*Bacteroidetes* (F/B) ratio affect metabolic processes (Chen et al., 2020), with a notable reduction in *Prevotella*—a key member of the *Firmicutes* phylum. *Prevotella* is a critical source of butyrate, which provides energy to colonic cells, reduces oxidative stress, and exerts anti-inflammatory effects through short-chain fatty acids (SCFAs) that promote regulatory T cell activation. This mechanism helps reduce inflammation in both the intestine and other organs, inhibits immune cell adhesion, proliferation, and translocation, and modulates cytokine secretion (e.g., IL-6), thereby maintaining intestinal homeostasis and barrier integrity. The dysbiosis observed in psoriasis disrupts this balance, contributing to chronic inflammation and further damage to the intestinal epithelial barrier (Sitkin and Pokrotnieks, 2019; Stefia et al., 2020; Olejniczak-Staruch et al., 2021). Additionally, *Akkermansia muciniphila* plays a significant role in increasing the thickness of the intestinal mucus layer, enhancing intestinal barrier integrity, and preventing systemic inflammatory diseases such as IBD and atherosclerosis (Zhang et al., 2025). A significant reduction in *Akkermansia muciniphila* in patients with psoriasis weakens this protective mucus layer, exacerbating intestinal barrier dysfunction (Sinha et al., 2021). Similarly, fecal samples from psoriasis and PsA individuals have shown a marked decrease in the abundance of *Akkermansia* spp., *Ruminococcaceae* spp., and *Pseudobutyrivibrio* spp. (Myers et al., 2019). Overall, gut microbiota dysbiosis leads to an increase in opportunistic pathogens, which disrupt tight junctions between intestinal epithelial cells by promoting the secretion of inflammatory cytokines. This results in increased intestinal permeability, triggering the excessive activation of Th1 and Th17 cells while reducing Treg cell populations. The imbalance between Th17 and Treg cells further amplifies pro-inflammatory cytokine production, worsening intestinal barrier damage and facilitating the entry of more bacteria, metabolites, and toxins into the bloodstream. This positive feedback loop intensifies chronic inflammatory responses both in the skin and systemically (Thye et al., 2022). In conclusion, disruption of the intestinal barrier plays a pivotal role in triggering local or systemic immune reactions, marking it as a critical factor in the pathogenesis of chronic inflammatory diseases (Figure 2). Consequently, maintaining the integrity of the intestinal barrier has emerged as a key focus for understanding the etiology of psoriasis.

### 3.3 The impact of gut microbiota-related metabolites on psoriasis

**Figure 3:**
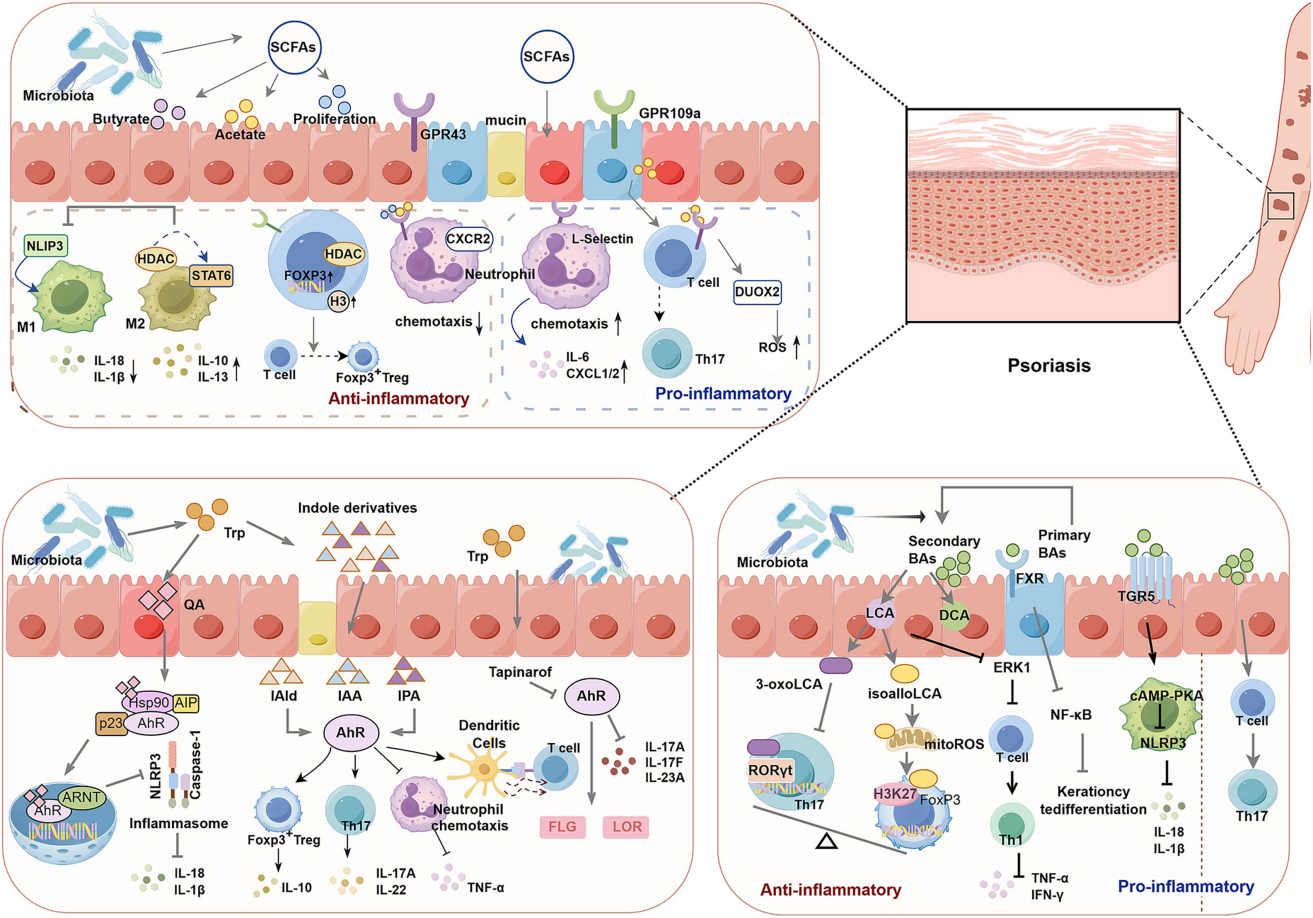
Schematic diagram of the role of intestinal flora metabolites in psoriasis. Short-chain fatty acids (SCFAs, such as butyrate and acetate): Metabolized by gut microbiota, they exert bidirectional effects through receptors like GPR43 and GPR109a. On one hand, they promote anti-inflammatory responses: inhibiting HDAC (histone deacetylase), activating pathways like STAT6 and FOXP3, enhancing Treg (regulatory T cell) function, and suppressing Th17 differentiation. On the other hand, they participate in pro-inflammatory processes: regulating chemotaxis of neutrophils and T cells (e.g., via CXCR2 and L-selectin pathways), inducing ROS production in Th17 cells, and amplifying skin inflammatory signals. Tryptophan (Trp) derivatives: Gut microbiota metabolize Trp into indole derivatives (e.g., IAld, IAA, IPA) or quinolinic acid (QA), activating the AhR (aromatic hydrocarbon receptor). On one hand, indole derivatives modulate dendritic cell, T cell, and neutrophil functions via AhR, influencing cytokine release (e.g., IL-17, IL-22) while regulating skin barrier proteins (FLG, LOR). On the other hand, QA activates AhR to trigger NLRP3 inflammasomes, releasing proinflammatory factors like IL-1β and IL-18, thereby driving inflammatory cascades. Bile acids (BAs): Classified into primary and secondary bile acids, they mediate distinct signaling pathways via FXR and TGR5 receptors. Secondary BAs (e.g., LCA, DCA) inhibit RORγt (Th17 transcription factor) and modulate HDAC activity to exert anti-inflammatory effects; however, certain BAs also activate NF-κB, NLRP3, or influence the cAMP-PKA pathway, promoting Th1 and Th17 differentiation while inducing abnormal keratinocyte differentiation, thereby exacerbating psoriatic skin lesions. Through the complex regulatory network of the “gut-skin axis,” imbalances in these metabolites disrupt immune homeostasis and skin barrier function, ultimately driving the onset and progression of psoriasis.

Gut microbiota metabolites include various substances such as SCFAs, tryptophan (Trp) metabolites, bile acids (BAs), LPS, phenols, and phenolic acids (Sipos et al., 2021). These microbe-derived metabolites play a pivotal role in modulating host immune function, regulating both local and systemic immune balance, maintaining intestinal barrier integrity, and alleviating psoriasis and its complications (Figure 3) (Zhang et al., 2024).

#### 3.3.1 SCFAs

SCFAs are essential microbial metabolites primarily produced by beneficial gut microbiota. The three most common SCFAs—acetate, propionate, and butyrate—are mainly produced through the fermentation of *Bacteroidetes* (acetate and propionate) and *Firmicutes* (butyrate) (Koh et al., 2016; Sipos et al., 2021). In psoriasis, SCFAs can act directly on target sites or through specific receptors to help maintain immune homeostasis. Notably, SCFAs exhibit biphasic regulatory effects in psoriasis.

On one hand, SCFAs exhibit anti-inflammatory properties. Psoriatic lesions are characterized by an increased number of pro-inflammatory (M1) macrophages and a decrease in anti-inflammatory (M2) macrophages (Shao et al., 2021). SCFAs, particularly butyrate, inhibit M1 macrophage activation and the secretion of pro-inflammatory mediators (e.g., IL-18 and IL-1β) by negatively regulating the NLRP3-mediated inflammatory signaling pathway, thereby reducing inflammation. Additionally, SCFAs promote STAT6 signaling and suppress histone deacetylases (HDACs), facilitating M2 macrophage polarization through histone acetylation (Ji et al., 2016; Mali et al., 2010). Although the precise mechanisms by which SCFAs regulate psoriatic macrophages are not fully understood, their role in macrophage polarization may offer a mechanism for improving psoriatic lesions. Additionally, forkhead box protein P3 (Foxp3), a key regulator of Treg cells, is essential for Treg development and function (Hori et al., 2003). As HDAC inhibitors, SCFAs promote the acetylation of histone H3 on the Foxp3 gene, which induces naive CD4^+^ T cells to express Foxp3 and differentiate into induced regulatory T cells (pTregs) (Sun et al., 2018). Furthermore, SCFAs, as HDAC inhibitors, significantly suppress TNF-α and IL-6 expression, alleviating systemic inflammation (Rooks and Garrett, 2016). They also stimulate the expression of filaggrin and transglutaminase-1, promoting the terminal differentiation of epidermal KCs to maintain skin homeostasis (Leon et al., 2014; Eslick et al., 2022). Moreover, SCFAs mitigate inflammation by inhibiting neutrophil recruitment to inflamed sites. Oral administration of acetate reduces C-X-C motif chemokine receptor 2 (CXCR2) expression in neutrophils and affects their chemotaxis *via* the G protein-coupled receptor GPR43, thereby alleviating skin inflammation (Maslowski et al., 2009; Wang et al., 2020; Wang and Jin, 2020).

On the other hand, SCFAs, particularly acetate and propionate, can exert pro-inflammatory effects by inducing neutrophil chemotaxis through GPR43 activation and upregulating the expression of the pro-inflammatory cytokine IL-6 and chemokines such as CXCL1/2. This mechanism may worsen psoriatic skin lesions (Krejner et al., 2018). For instance, acetate administration in psoriatic mice increases Th17 immune responses and dual oxidase-2 (DUOX2) expression, exacerbating skin inflammation (Nadeem et al., 2017). Current studies suggest that GPR43 activation may intensify inflammation in psoriasis, whereas acetate shows marked anti-inflammatory effects in the absence of GPR43 (Nadeem et al., 2017). These findings indicate that acetate’s pro-inflammatory effects are GPR43-dependent. Additionally, SCFAs can activate Tregs *via* GPR109a, inhibiting excessive Th17 cell activation and reducing inflammation in psoriasis (Schwarz et al., 2023). Clearly, SCFAs—as key metabolites of the gut microbiota—exhibit variable effects in inflammation regulation. Their anti-inflammatory or pro-inflammatory bias is jointly modulated by local concentrations, receptor expression patterns, and microenvironmental characteristics. At physiological concentrations, SCFAs primarily function as potent anti-inflammatory agents. However, in patients with psoriasis and psoriatic arthritis (PsA), the abundance of SCFA-producing bacteria (such as *Faecalibacterium prausnitzii* and *Akkermansia muciniphila*) significantly reduced in patients with psoriasis and PsA, leading to insufficient synthesis of SCFAs like butyrate. This not only fails to effectively induce Treg differentiation to suppress Th17 cell activation but also disrupts the tight junctions of the intestinal epithelium. Consequently, LPS enters the bloodstream, activating systemic inflammatory responses and ultimately exacerbating joint and skin inflammation through the “gut-joint axis” or “gut-skin axis” (Nadeem et al., 2017; Bonomo et al., 2025). Furthermore, the anti-inflammatory effects of SCFAs depend on the expression and activation of their specific receptors. When target cells highly express SCFA-specific receptors, SCFAs can efficiently activate anti-inflammatory pathways. However, when SCFA receptors are downregulated, SCFAs exert pro-inflammatory effects by upregulating pro-inflammatory cytokine secretion, indirectly promoting the formation of psoriatic plaques (Nadeem et al., 2017; Krejner et al., 2018). Genetic polymorphisms in short-chain fatty acid receptors (such as GPR43 and GPR41) may also influence inflammatory outcomes and metabolic processes (Ichimura, 2017). Moreover, SCFA effects are not isolated but interact closely with the inflammatory state, microbiota composition, and other inflammatory mediators within the local microenvironment. A stable microenvironment can enhance SCFAs’ anti-inflammatory effects, whereas inflammatory microenvironments (such as psoriasis, PsA, or IBD) may counteract their anti-inflammatory actions through “pro-inflammatory factor overload.” These seemingly contradictory actions highlight the complexity of immune regulation, further underscoring the critical role of short-chain fatty acids in maintaining overall health and immune equilibrium.

#### 3.3.2 Trp

Trp, an essential amino acid, plays a pivotal role in protein synthesis and serves as a precursor for various bioactive compounds. Its metabolic pathways primarily involve the kynurenine (Kyn), 5-hydroxytryptamine, and indole routes (Oxenkrug and Navrotska, 2023). Among these, the Kyn pathway is the primary degradation route for Trp. The gut microbiota directly metabolizes Trp into indoles and indole derivatives [e.g., indole-3-aldehyde (IAld), indole-3-acetic acid (IAA), indole-3-propionic acid (IPA)], tryptamine, and other products. These metabolites act as ligands for the aryl hydrocarbon receptor (AhR), which plays a vital role in immune homeostasis and intestinal barrier function (Lopez et al., 2021).

Clinical studies have confirmed significant imbalances in Trp metabolism in the peripheral blood and skin lesion tissues of psoriasis patients: On one hand, levels of protective indole metabolites with AhR-activating activity (e.g., IPA, IAld) are markedly reduced. On the other hand, levels of pro-inflammatory Kyn pathway metabolites [e.g., Kyn, quinolinic acid (QA)] are elevated. Moreover, the severity of this imbalance positively correlates with psoriasis area and PASI scores (Chen et al., 2025; Gasaly et al., 2021). Further mechanistic studies revealed that metabolites influence psoriasis progression through the AhR pathway, including effects on intestinal and skin barrier integrity, as well as immune cell regulation (Gasaly et al., 2021; Li et al., 2024; Stone and Williams, 2023; Chen et al., 2025). AhR binding to its ligands significantly impacts the differentiation and proliferation of Th17 and Treg cells (Gasaly et al., 2021). Specifically, AhR agonists promote the production of IL-17A and IL-22 during Th17 cell differentiation, while Th17 polarization is markedly inhibited in AhR-deficient T cells. Additionally, AhR regulates immune responses by promoting Treg cell polarization and IL-10 secretion, suppressing excessive inflammation (Ehrlich et al., 2018; Lopez et al., 2021). Research confirms that Kyn promotes Treg cell differentiation through AhR, suppresses Th17 cell effector functions, and upregulates PD-1 expression on T cell surfaces, thereby exacerbating T cell exhaustion. Concurrently, it enhances the pro-inflammatory phenotype of keratinocytes, leading to increased release of IL-6 and IL-8 (Miyamoto et al., 2024). Similarly, a recent study established a mouse model carrying the human psoriasis-associated mutation Card14E138A/+, confirming that indole-producing gut bacteria promote the accumulation of the host metabolite indole-3-sulfate (I3S), activate the AhR to remodel the epigenetic state of skin Th17 cells and enhance their effector functions, ultimately remotely driving psoriatic inflammation (Wang et al., 2025).

At the level of innate immunity, Zhu et al. (2020) demonstrated in AhR gene-knockout mice that AhR alleviates psoriatic skin lesions by inhibiting neutrophil recruitment and reducing the expression of inflammatory cytokines (e.g., TNF-α, IL-17). AhR also plays a critical role in DC maturation and function. Activated AhR promotes DC maturation, enhances their antigen-presenting capacity, and facilitates the transfer of pathogen-related information to T cells, initiating adaptive immune responses. Endogenous AhR ligands, such as FICZ, improve skin inflammation in psoriasis by inducing CYP1A1 expression and reducing the transcription/expression of psoriasis-related genes. Moreover, the Kyn metabolite QA inhibits NLRP3 inflammasome activation and the secretion of IL-1β/IL-18 *via* AhR nuclear translocation, thus suppressing epidermal hyperplasia and inflammatory infiltration in psoriatic mice. In psoriatic lesions, QA levels showed a negative correlation with IL-1β expression and a negative correlation with PASI scores (Qiao et al., 2022).

A multicenter clinical study confirmed that topical application of the AhR agonist Tapinarof significantly reduces IL-17A/F and IL-23A protein levels in psoriatic lesions, enhances antioxidant responses, and modulates skin barrier proteins (e.g., filaggrin [FLG], loricrin [LOR]) to alleviate psoriasiform skin lesions (Bissonnette et al., 2021). Similarly, AhR agonists demonstrated favorable therapeutic effects in an imiquimod (IMQ)-induced mouse model (Colonna, 2014), while the AhR antagonist CH-223191 exacerbates psoriasis-related gene expression in patient biopsies by blocking the AhR pathway (Um et al., 2020). These findings suggest that AhR is a promising therapeutic target for psoriasis. While some of AhR’s functions in psoriasis have been validated, further research is required to fully understand the molecular mechanisms by which AhR regulates skin homeostasis during inflammatory responses.

#### 3.3.3 BAs

BAs, produced in the liver and further modified in the intestine, play a pivotal role in lipid and fat-soluble vitamin digestion and absorption (Perino and Schoonjans, 2022). Cholesterol is converted into primary BAs, which are then metabolized by gut microbiota in the distal small intestine and colon to form secondary BAs, such as deoxycholic acid (DCA) and lithocholic acid (LCA) (Fiorucci et al., 2021a,b). BAs act as signaling molecules, activating specific receptors to regulate cellular functions and metabolic processes. These receptors include membrane receptors like TGR5 (GPBAR1) and nuclear receptors, such as the farnesoid X receptor (FXR) and vitamin D receptor (VDR) (Fiorucci et al., 2021a,b). Activation of these receptors influences glucose and lipid metabolism and helps maintain overall health by modulating immune system activity (Yoon et al., 2023).

Gut microbiota-modified BA metabolites exert complex bidirectional effects in psoriasis. Studies have shown that serum BA levels are significantly reduced in patients with psoriasis compared to healthy controls (Sorokin et al., 2018; Paine et al., 2023). Two LCA derivatives identified in humans and rodents have pronounced anti-inflammatory effects on CD4^+^ T cells. 3-oxoLCA inhibits Th17 cell differentiation by interacting with ROR*γ*t, thereby suppressing its transcriptional activity. On the other hand, isoalloLCA promotes Treg cell differentiation by stimulating oxidative phosphorylation (OXPHOS), increasing mitochondrial reactive oxygen species (mROS), and enhancing Foxp3 expression *via* histone (H3K27) acetylation at the Foxp3 promoter (Hang et al., 2019). Interestingly, the regulatory effects of LCA derivatives on CD4^+^ T cells may occur independently of commensal bacteria. Further research indicates that LCA inhibits CD4^+^ T cell differentiation into Th1 cells by blocking ERK1 phosphorylation, which in turn downregulates the expression of TNF-α and IFN-γ cytokines (Pols et al., 2017). Cytokines like TNF-α and IFN-γ are known to stimulate KC proliferation, induce chemokine and cytokine production, and promote psoriasis progression (Armstrong and Read 2020). Animal studies by Chen et al. have demonstrated that gut microbiota supplementation enhances BA production, reduces inflammation through FXR signaling pathway regulation, inhibits KC proliferation, and improves skin barrier function, thereby alleviating psoriasis symptoms (Chen et al., 2023). Additionally, secondary BAs exert anti-inflammatory effects by activating TGR5, which initiates cyclic adenosine monophosphate (cAMP)-mediated inhibition of the NLRP3 inflammasome and NF-κB signaling pathways (Zhao et al., 2023a). However, elevated levels of certain secondary BAs promote Th17 cell differentiation, exacerbating skin inflammation (Sorrentino et al., 2020). Although the role of BAs in psoriasis remains incompletely understood and their therapeutic efficacy requires further validation, it is clear that BAs are crucial mediators through which gut microbiota influence psoriasis progression, warranting additional investigation.

In addition to metabolites from beneficial intestinal microbiota, LPS, a major component of gram-negative bacteria, plays a pivotal role in inducing the overexpression of pro-inflammatory cytokines such as TNF-α, IL-6, and IL-8, thereby promoting a moderate inflammatory state *in vivo* (López-Moreno et al., 2017; Rorato et al., 2017). Polyamines, including spermine and spermidine, produced by gut microbiota, are also significant in the pathogenesis of psoriasis. Recent studies show that in IMQ-induced psoriasiform mouse models, spermine and its derivative SD1 act as immunosuppressive agents, influencing T cell metabolic reprogramming. These compounds effectively inhibit CD45^+^ immune cell infiltration in the dorsal skin and ears, limit splenomegaly, and reduce epidermal hyperplasia. Notably, spermine derivative 1 (SD1) induces more potent remission of psoriatic skin lesions than spermine itself (Xu et al., 2024). Neuwirth et al. (2025) analyzed tissue samples from patients with psoriasis, cutaneous sarcoidosis, and ulcerative colitis, revealing reduced Treg cell numbers with pro-inflammatory phenotypes and significantly upregulated spermidine/spermine acetyltransferase 1 (SSAT1) gene expression. CRISPRa-mediated overexpression of SSAT1 led to a loss of Treg cell function, impairing their ability to suppress effector T cells and promoting pro-inflammatory cytokine secretion. These findings highlight potential new therapeutic targets and strategies for managing chronic inflammatory skin diseases. Overall, psoriasis progression is closely linked to alterations in gut microbiota, with microbiota-derived metabolites acting as key mediators of these effects.

SCFAs, Trp, and BAs, as core metabolites of the gut microbiota, do not function independently but interact synergistically to jointly regulate host immune homeostasis, metabolic health, and barrier function. Among these, SCFAs can inhibit the activity of hepatic cholesterol 7*α*-hydroxylase (CYP7A1), thereby reducing primary bile acid (BA) synthesis and consequently affecting the production of secondary BAs such as deoxycholic acid (DCA) and lithocholic acid (LCA). LCA derivatives (e.g., CYP7A1) activity to reduce primary bile acid (BA) synthesis, thereby influencing the production of secondary BAs (e.g., deoxycholic acid DCA, lithocholic acid LCA). LCA derivatives (e.g., 3-oxoLCA, isoallo LCA) can then modulate Th17/Treg balance by regulating RORγt and Foxp3 expression (Hang et al., 2019; Zhang et al., 2024). Furthermore, SCFAs may enhance AhR expression in intestinal epithelial cells, promoting the binding of tryptophan metabolites (e.g., IPA) to AhR. This synergistically inhibits NLRP3 inflammasome activation, collectively alleviating skin inflammation (Qiao et al., 2022; Chen et al., 2025). Research on the interactions among gut microbiota metabolites remains scarce. Future studies should aim to construct metabolic interaction networks to provide more systematic mechanistic models, thereby deepening our understanding of the gut microbiota-metabolite-host immune axis.

## 4 Therapeutic strategies based on gut microbiota

### 4.1 Probiotics/prebiotics/synbiotics

Probiotics, live microorganisms beneficial to human health, help the body resist foreign pathogen invasion and inhibit harmful bacterial proliferation. They exert positive effects through mechanisms such as inflammation suppression, immune response modulation, and the rebalancing of gut microbial composition (Alesa et al., 2019; Lu et al., 2021). Currently, *Lactobacillus* and *Bifidobacterium* preparations are the most widely used (Thye et al., 2022). Probiotics have been shown to improve psoriasis: although the contents and usage of probiotic supplements vary across studies, both animal experiments and human trials confirm their significant therapeutic effects in psoriasis (Chen et al., 2017; Szántó et al., 2019).

Navarro-López et al. (2019) found that administering probiotics (*B. longum*, *B. lactis*, and *L. rhamnosus*) induced changes in the gut microbiome, including decreases in *Rhodococcus* and *Micromonospora* species and increases in *Collinsella* and *Lactobacillus* species. These changes were correlated with alterations in skin and intestinal Th17 cells, which play a central role in psoriatic pathogenesis. Additionally, probiotics such as *B. adolescentis CCFM667*, *Limosi Lactobacillus reuteri CCFM1132*, and *Lacticaseibacillus paracasei CCFM1074* restore gut microbial balance in psoriatic mice by reducing the relative abundance of *Rikenellaceae* (Lu et al., 2021).

Beyond modifying gut microbiota composition in psoriasis treatment, probiotics can directly modulate immunity. In IMQ-induced psoriasis mice, *Lactiplantibacillus plantarum GMNL-77* reduced the proportion of IL-17A^+^CD4^+^ T cells and decreased the expression of inflammatory factors such as TNF-α, IL-23, and IL-17A, leading to amelioration of erythematous and scaly lesions (Chen et al., 2017). Similarly, oral administration of *B. breve CCFM683* (dose >108.42 CFU/day) significantly improved psoriasis in mice through multifaceted mechanisms: reducing inflammatory cytokines, regulating KC proliferation/differentiation, protecting the epidermal barrier by increasing filaggrin and loricrin, and promoting BA production. However, the dosage conversion between mice and humans remains unclear, necessitating future clinical trials to assess *CCFM683*’s efficacy in patients with psoriasis (Chen et al., 2023).

A recent study further demonstrated that *Bifidobacterium longum* treatment maintains intestinal barrier integrity in mice by increasing propionate levels, regulating the Th17/Treg cell balance, reducing the expression of IL-6, TNF-α, and IL-23A in intestinal tissues, and promoting IL-10 secretion. Combined use of *Bifidobacterium longum* and methotrexate (MTX) enhanced MTX’s therapeutic efficacy in psoriasis (Mao et al., 2025). Several clinical trials have similarly shown that probiotics alleviate psoriasis symptoms and reduce disease flares. In randomized controlled trials (RCTs), patients treated with probiotics such as Lactobacillus and Bifidobacterium experienced improved PASI scores and reduced levels of inflammatory markers, including CRP, IL-6, and TNF-α (Buhaș et al., 2023; Navarro-López et al., 2019). Notably, mixed probiotic treatments also reduced the risk of psoriasis recurrence, suggesting that oral probiotics may offer lasting therapeutic effects (Navarro-López et al., 2019). These findings reinforce the connection between gut microbiota and psoriasis and provide novel clinical intervention strategies for treating the condition.

Prebiotics, which serve as substrates for probiotics, selectively promote the growth, reproduction, and metabolic activity of beneficial gut microbiota, such as Bifidobacterium and Lactobacillus. Common prebiotics include inulin, oligogalactose (GOS), oligofructose (FOS), and xylooligosaccharides (XOS) (Gibson et al., 2017; Markowiak and Śliżewska, 2017; Yadav et al., 2022). Patients with psoriasis receiving prebiotic supplements (FOS, XOS, and GOS) exhibit better outcomes in disease activity indices, including the psoriasis area and severity index (PASI), dermatologic quality of life index (DLQI), inflammatory markers, and skin thickness, compared to those not receiving prebiotics (Buhaș et al., 2023). One key mechanism by which prebiotics influence psoriasis is through cytokine balance, which modulates immune function. For example, sustained GOS intake for 10 weeks significantly reduced pro-inflammatory cytokines IL-1, IL-6, and TNF-α while increasing anti-inflammatory IL-10, leading to improved inflammatory symptoms of psoriasis (Shokryazdan et al., 2017; Vulevic et al., 2008). Additionally, prebiotics stimulate the production of intestinal SCFAs, inhibit intestinal inflammation, and maintain intestinal mucosal integrity after fermentation by beneficial gut bacteria. SCFAs also possess anti-inflammatory and antioxidant properties, helping to alleviate common metabolic disorders in psoriasis, such as hyperuricemia and hyperlipidemia (Sobh et al., 2022).

Synbiotics, a combination of probiotics and prebiotics, enhance host health by synergistically promoting the colonization and proliferation of beneficial intestinal bacteria. Due to their role in immune regulation and anti-inflammatory responses, synbiotics are used in the management of various chronic diseases (Moravejolahkami et al., 2023). Three months of continuous synbiotic intake significantly increases the abundance of beneficial microbiota, including *Bifidobacteria*, *Lactobacilli*, *Clostridia*, and *Bacilli* (Sergeev et al., 2020; Álvarez-Arraño and Martín-Peláez, 2021). In a psoriasis clinical trial, patients treated with a combination of prebiotics (FOS, XOS, and GOS) for 8 weeks and oral probiotics for 12 weeks showed significant reductions in inflammatory markers, leading to marked improvement in psoriasis outcomes (Suriano et al., 2023). Similarly, Buhaș et al. (2023) assessed the efficacy of a probiotic and prebiotic mixture in psoriasis treatment. Results indicated significant improvements in PASI scores, DLQI, inflammatory markers, and lesion thickness in treated patients compared to controls. In conclusion, probiotics, prebiotics, and synbiotics can complement traditional psoriasis treatments by targeting inflammation and gut microbiota imbalances. However, therapeutic efficacy remains inconsistent, and further research is required to confirm their safety and efficacy in psoriasis treatment, as well as to determine the optimal species combinations, dosages, and treatment durations.

### 4.2 Fecal microbiota transplantation

A healthy gut microbiota plays a pivotal role in balancing the immune system, enhancing intestinal barrier function, regulating the “gut-skin axis,” and producing beneficial metabolites, all of which help alleviate skin inflammation and promote repair. However, for psoriasis individuals with severe microbiota dysbiosis, relying solely on probiotics or prebiotics may not fully restore intestinal ecology. Thus, fecal microbiota transplantation (FMT) has emerged as a potential therapeutic option. FMT involves screening feces from healthy donors (HD) and transplanting the selected microbial community into the patient’s gastrointestinal tract, thereby improving gut microbial composition and remodeling the intestine (Li et al., 2016; Gu et al., 2023).

Numerous studies have demonstrated the efficacy of FMT in improving psoriasis. A mouse study comparing feces from HD and psoriatic patients (PSD) found that HD-derived FMT prevented Treg/Th17 imbalance in psoriatic mice, with significant enrichment of *Lactobacillus reuteri* in both the fecal and skin microbiomes. Notably, *L. reuteri* supplementation inhibited pro-inflammatory pathways and enhanced skin barrier integrity. In contrast, PSD-derived FMT exacerbated IMQ-induced psoriasis symptoms in mice (Chen et al., 2021). Another experiment showed that PSD-derived FMT worsened psoriasiform skin inflammation in mildly symptomatic mice by increasing Th17 infiltration/differentiation, elevating *Prevotella* abundance, reducing beneficial *Parabacteroides distasonis*, and altering gut microbiota composition (Zhao et al., 2023b).

While *in vitro* studies support the role of FMT in psoriasis, clinical evidence remains mixed. The first clinical case report of FMT in a patient with severe plaque psoriasis with concurrent IBS showed significant reductions in serum TNF-α levels and PASI scores after two FMT sessions, along with improved histology and intestinal symptoms (Gu et al., 2023). Another case report documented a patient with PsA treated with FMT for *Clostridium difficile* infection, who experienced reduced PsA activity, decreased joint pain and swelling, and improved skin lesions (Selvanderan et al., 2019). These studies highlight the potential benefits of FMT for psoriasis and PsA. However, a randomized placebo-controlled trial involving 31 patients with PsA found that endoscopy-guided FMT did not improve psoriasis symptoms compared to sham procedures, despite higher treatment response rates in the FMT group (Kragsnaes et al., 2021).

Differences in FMT efficacy for psoriasis may be associated with variations in donor selection. Existing studies employ inconsistent donor screening criteria, with some utilizing healthy donors while others fail to rigorously exclude potential gut microbiome abnormalities (such as subclinical inflammation) in donors. This leads to significant disparities in post-transplant microbial colonization outcomes (Gu et al., 2023). Additionally, microbial compatibility between donors and recipients may influence treatment efficacy. For instance, donors enriched with *Ruminococcus* may be more suitable for psoriasis patients exhibiting markedly reduced gut microbial diversity (Myers et al., 2019). Similarly, administration routes play a crucial role: upper gastrointestinal delivery (e.g., via gastroscopy) facilitates microbial colonization in the small intestine, making it suitable for improving inflammation related to intestinal barrier function. Conversely, lower gastrointestinal delivery (e.g., via colonoscopy) promotes colonization in the colon, potentially offering greater efficacy for diseases regulated by colonic metabolites (e.g., SCFAs). Differences in microbial colonization sites resulting from varying administration routes may represent a key reason for the apparent contradiction between the findings of Myers et al. (2019), Kragsnaes et al. (2021), and Gu et al. (2023). Most clinical studies did not employ antibiotics or bowel cleansing agents for pre-treatment, resulting in ineffective clearance of the recipient’s original pathogenic bacteria and compromising the colonization efficiency of the donor microbiota. An animal study confirmed that pre-treatment significantly enhances FMT’s efficacy in improving psoriasiform inflammation, highlighting the importance of optimizing pre-treatment protocols in clinical research (Chen et al., 2021). Furthermore, the sustained colonization of the transplanted microbiota post-FMT is crucial for maintaining therapeutic effects. Unfortunately, existing studies lack long-term follow-up data, yet this gap also points the way forward for future FMT research. Undeniably, FMT’s ability to enrich beneficial gut microbiota, modulate inflammatory pathways, and remodel the intestinal barrier provides a strong theoretical foundation for its use in immune-mediated diseases like psoriasis. Nevertheless, FMT for psoriasis remains in the exploratory phase, lacking sufficient clinical data. Future research must further investigate FMT’s specific efficacy and mechanisms in psoriasis to offer new therapeutic hope for patients.

### 4.3 Other promising treatments

Infections can trigger or worsen psoriasis, particularly acute pitting psoriasis, and are often linked to microorganisms such as bacteria, fungi, and viruses (Tsai and Tsai, 2019). Consequently, antibiotics are typically the treatment of choice for infectious psoriasis, with penicillins and macrolide antibiotics commonly used as first-line therapies. The antibacterial spectrum of macrolide antibiotics includes *Staphylococcus*, *Streptococcus*, and *Enterococcus* (Tsai and Tsai, 2019) —bacteria that are significantly increased in the intestines of patients with psoriasis (Chen et al., 2020). Oral azithromycin significantly improves skin rash in patients with psoriasis. Mechanistic studies suggest that azithromycin acts by interfering with TLRs to reduce the expression of inflammatory factors in psoriasis mice (Saxena and Dogra, 2010). Similarly, erythromycin has been shown to improve PASI scores in patients with psoriasis (Polat et al., 2007). However, antibiotic therapy may also be ineffective or even exacerbate psoriasis. For example, a group of psoriasis individuals with active *streptococcal* throat infections did not experience a significant reduction in PASI scores after receiving antibiotics along with standard topical psoriasis treatments, compared to patients without active infections (Bonciani et al., 2025). Additionally, tetracycline antibiotics have been shown to exacerbate plaque psoriasis. Long-term, large-scale use of antibiotics may reduce gut microbiota diversity and promote the growth of drug-resistant strains (Tsai and Tsai, 2019). Further studies are needed to determine the optimal dosage and duration of antibiotic therapy for treating infectious psoriasis.

An emerging frontier in psoriasis therapy is the selective reduction of pathogenic bacteria and rebalancing of the microbiota using bacteriophages (phages). A microecological study of psoriasis revealed distinct phage compositions between lesional skin, contralateral non-lesional skin, and healthy control skin. Samples with higher phage species richness exhibited suppressed abundances of host bacteria, suggesting that phages could serve as effective therapeutic agents by replenishing deficient phages in skin lesions and potentially correcting bacterial dysbiosis (Nong et al., 2024; Wang et al., 2020). Furthermore, in skin infections, phage supplementation targeting *Pseudomonas aeruginosa* significantly reduced bacterial abundance at lesion sites, demonstrating that phage therapy can mitigate inflammation and rebalance dysbiotic gut microbiota (Vieira et al., 2012).

Leveraging phages’ high specificity, researchers have developed a cocktail of five wild-type phages targeting *Acinetobacter baumannii*. This formulation reduced biological burden and pro-inflammatory cytokine levels in infected mouse wounds while decreasing infection-related morbidity (Regeimbal et al., 2016). These findings position phage and phage cocktail therapies as promising strategies for clinical psoriasis management. Future research may explore combinatorial approaches with FMT and biologic agents (e.g., IL-23 inhibitors) to address current therapeutic challenges and improve outcomes for patients who are unresponsive to existing treatments.

## 5 Conclusion and prospects

In recent years, accumulating studies have revealed a close connection between gut microbiota and psoriasis, establishing the “gut-skin axis” as a central factor in the disease’s pathogenesis. In patients with psoriasis, intestinal flora diversity is significantly reduced, with notable alterations in microbiota structure, such as an imbalanced FB ratio, a decrease in beneficial genera like *Akkermansia* and *Faecalibacterium*, and an increase in pro-inflammatory genera such as *Ruminococcus* and *E. coli*. These dysbiotic states contribute to psoriatic pathogenesis through several mechanisms: disrupting intestinal barrier integrity, which allows pro-inflammatory substances (e.g., LPS) to enter the bloodstream and activate systemic inflammatory cascades; disturbing the Treg/Th17 axis balance, leading to immune dysregulation; and modulating the production of key metabolites (e.g., SCFAs, Trp metabolites, BAs) that influence immune cell differentiation and skin barrier function. Additionally, the gut microbiota can serve as biomarkers for diagnosing and monitoring psoriasis. Specifically, *Akkermansia* and *Faecalibacterium* can be used as indicators for assessing disease activity, with studies confirming that reduced abundance of both is significantly correlated with psoriasis PASI scores (Tan et al., 2018; Sinha et al., 2021; Effendi et al., 2022). Serum expression levels of Claudin-3 and I-FABP can serve as quantitative indicators of intestinal barrier integrity, with elevated levels predicting the risk of psoriasis recurrence (Sikora et al., 2019; Lai et al., 2023).

Intervention strategies targeting gut microbiota have shown significant clinical promise. Probiotics, prebiotics, and synbiotics alleviate psoriasis symptoms by remodeling microbiota composition, strengthening intestinal barriers, and inhibiting pro-inflammatory pathways. FMT has reduced skin inflammation in animal models by restoring microbiota balance, and while clinical evidence remains mixed, it represents a new approach for reshaping the host’s immune microenvironment. Emerging strategies, such as phage therapy and precision antibiotic interventions, offer additional innovative directions for microbiota-targeted psoriasis treatment.

Clearly, interventions targeting the microbiome hold significant therapeutic potential. However, overcoming the high heterogeneity and complexity of the gut microbiome among individual patients to precisely identify pathogenic microbial features or beneficial microbial deficiencies remains a major challenge. The human microbiome is unique to each individual, and this variability leads to differing responses to treatment among patients, compelling us to develop personalized therapeutic strategies (Ejtahed et al., 2023). To establish standardized protocols and long-term efficacy for prebiotic, probiotic, and FMT interventions while ensuring consistent and reproducible outcomes, future clinical research should focus on large-scale, standardized randomized controlled trials. Concurrently, to fully understand the potential of gut microbiome-based therapies in psoriasis, future studies should enroll psoriasis patient subgroups exhibiting specific dysbiosis phenotypes (e.g., marked reduction in *Akkermansia*, increased *Firmicutes/ Bacteroides* ratio) and systematically monitor pre- and post-intervention dynamics in their microbiota composition, metabolites (e.g., SCFAs, LPS), and intestinal barrier integrity markers (e.g., Claudin-3, I-FABP). This approach will clarify efficacy associations in target populations and mitigate outcome biases stemming from patient microbiota heterogeneity. Furthermore, combining probiotics, prebiotics, and FMT in personalized treatment regimens may offer a more effective and synergistic approach for psoriasis management.

Currently, biologics remain the dominant treatment for psoriasis. Growing clinical evidence indicates that biologics can alter the microbial diversity of psoriasis patients (Bai et al., 2019; Valentini et al., 2021). For instance, use of the IL-17 inhibitor secukinumab significantly increases levels of *Bacteroides stercoris* and *Parabacteroides merdae*, thereby modulating intestinal inflammatory responses (Yeh et al., 2019). Similarly, in psoriasis patients treated with usnulinumab—which targets IL-12 and IL-23—the abundance of *Firmicutes phylum* and *Enterobacteriaceae* family Panbacteria significantly increases (Di et al., 2021). These bacteria improve psoriasis symptoms by suppressing intestinal oxidative stress and regulating the Th17/Treg balance (Polkowska-Pruszyńska et al., 2020). While existing biologics precisely target immune-inflammatory pathways and significantly improve clinical symptoms, challenges persist, including individual response variability, long-term safety concerns, and relapse upon discontinuation. Given the promising outcomes from gut microbiota-based therapies in current studies, integrating microbiome-targeting strategies with biologics to reshape immune balance through synergistic “source regulation-precision anti-inflammation” holds promise as a key direction to overcome existing treatment limitations. Research in this field remains scarce, and these findings are preliminary, requiring more comprehensive studies for validation.

Despite the substantial progress made in understanding the relationship between gut microbiota and psoriasis, several key areas still require further exploration. Mechanistic studies are needed to better understand how microbiota-derived metabolites regulate immune cells in the gut-skin axis. In clinical practice, larger-scale studies are necessary to establish standardized protocols for gut microbiota interventions, such as FMT, and to develop biomarkers for microbiota metabolites to monitor disease progression. These efforts aim to provide new therapeutic strategies for patients with psoriasis.
